# Poly(lactic acid) (PLA) Based Tear Resistant and Biodegradable Flexible Films by Blown Film Extrusion

**DOI:** 10.3390/ma11010148

**Published:** 2018-01-17

**Authors:** Norma Mallegni, Thanh Vu Phuong, Maria-Beatrice Coltelli, Patrizia Cinelli, Andrea Lazzeri

**Affiliations:** 1 Department of Civil and Industrial Engineering, University of Pisa, Via Diotisalvi 2, 56122 Pisa, Italy; norma.mallegni@gmail.com (N.M.); maria.beatrice.coltelli@unipi.it (M.-B.C.); patrizia.cinelli@pi.ipcf.cnr.it (P.C.); 2 Department of Chemical Engineering, Can Tho University, 3/2 Street, Can Tho 90000, Vietnam; thanhvu@ctu.edu.vn; 3 National Research Council, Institute of Chemical Physical Processes, Via Moruzzi 1, 56124 Pisa, Italy

**Keywords:** biodegradable polymer blends, mechanical properties, blown film extrusion, plasticizer, nucleating agent

## Abstract

Poly(lactic acid) (PLA) was melt mixed in a laboratory extruder with poly(butylene adipate-*co*-terephthalate) (PBAT) and poly(butylene succinate) (PBS) in the presence of polypropylene glycol di glycidyl ether (EJ400) that acted as both plasticizer and compatibilizer. The process was then scaled up in a semi-industrial extruder preparing pellets having different content of a nucleating agent (LAK). All of the formulations could be processed by blowing extrusion and the obtained films showed mechanical properties dependent on the LAK content. In particular the tearing strength showed a maximum like trend in the investigated composition range. The films prepared with both kinds of blends showed a tensile strength in the range 12–24 MPa, an elongation at break in the range 150–260% and a significant crystallinity.

## 1. Introduction

The development of new materials derived from renewable sources is an objective of high priority on a technological and environmental point of view to reduce dependence on non-renewable sources (oil). Currently, the global consumption of plastics is more than 200 million tons, with an annual increase of approximately 5% [1]. Although the use of oil for plastics is assessed at about 4% of global consumption, it is anyway important to try to replace them with alternative raw materials. Until now petrochemical-based plastic have been used as packaging materials. This kind of petrochemical plastics are preferred because they show good mechanical performances, heat stability, good barrier properties, high availability, and a relative low cost. Nevertheless, their end-life management is often not easy, because only some plastic items, e.g., those made of only one material, can be selectively collected and recycled [2]. Plastic films can be recycled but they have a low apparent density. The packages consisting of different materials, e.g., multilayer packages, cannot be easily recycled, but they usually can neither be composted. 

The production of flexible films by using compostable plastics can be particularly important because they can be used for preparing low apparent-density packaging, such as pouches or bags, or multilayer packaging by coupling them with paper or board, thus producing fully compostable packaging. Anyway, for addressing this market, it is necessary to overcome the issue of low tear resistance of this kind of compostable films, and allow their production with conventional industrial processing methods, such as blown film extrusion.

PLA is an environmentally friendly polymer because it is compostable and it derives from renewable resources. It shows a high elastic modulus and a high transparency. PLA has also strong limitations: low glass transition temperature, low thermal stability, high brittleness, and low crystallization rate. 

The production of PLA presents numerous advantages, as it is renewable [3], its production consumes high quantities of carbon dioxide [4], it provides significant energy savings [5], it is recyclable and compostable [6,7], and the physical and mechanical properties can be partially tailored by properly selecting the polymer architecture [8,9]. PLA is currently used in rigid containers for water, juice and yogurt. In particular PLA based packaging is used in Europe, Japan and North America [10]. Currently it represents one of the cheapest bio-based and biodegradable alternative to petrochemical plastics.

To increase the flexibility of PLA it is possible to add a low molecular weight plasticizer [11,12] or blending it with a flexible polymer, such as poly(butylene adipate-*co*-terephthalate) (PBAT) [13,14]. In the latter case, the use of a proper compatibilizer was demonstrated to be important for a better modulation of properties thanks to the achievement of a phase morphology characterized by a lower dimension of the dispersed PBAT phase and an increased adhesion [15,16]. The addition to PLA of poly(butylene succinate) (PBS) was also reported for increasing the ductility of PLA based blends [17,18,19]. Recently, the addition of nano-calcium carbonate to PLA/PBS blends allowed for improving their properties maintaining an improved impact resistance, but combined with an improved flexural strength [20]. Interestingly, PLA/PBS blends with a PLA matrix were found to show a partial shape memory effect [21] that makes them interesting for producing films for secondary packaging. Furthermore, tricontinuous blends consisting in PLA, PBAT, and PBS (each at 33.3% by weight) were demonstrated to be promising for preparing biobased blends with properties similar to poly(ethylene) [22]. However, the phase distribution is usually not easy to be controlled and stabilized in ordinary industrial processing. Moreover, PLA based blends are more promising in terms of cost and environmentally friendly, as PLA is fully from renewable sources, whereas PBAT and PBS are partially synthesized, at least up to now, from non-renewable resources. PBS will soon be produced completely from renewable sources as plants for producing succinic acid from biomass have been developed [22]. In the future, also PBAT may be renewable, as the industrial synthesis of its monomers from renewable resources has been almost fully achieved [23].

An important issue in extrusion is the control of crystallization rate, usually low in PLA, as it influences the possibility to rapidly and efficiently cut the extruded strand, thus easily producing pellets. Moreover, in blown films extrusion, it much influences the process stability and the achievement of a stiff film. In order to increase the crystallization rate usually nucleating agents are added [24] and interesting synergistic effects were noticed by using both a plasticizer and a nucleating agent in PLA [25,26]. In particular, Fehri et al. [27] noticed that the use of oligomeric lactic acid as plasticizer and LAK301 (an aromatic sulfonate derivative) as nucleating agent, were much more efficient in decreasing the half crystallization time of PLA, and, by applying a design of experiment approach, they identified the best composition range where the half crystallization time was minimized.

In applications such as flat die extrusion or blow extrusion, it is important the fine tuning of melt viscosity and melt strength. In these cases, the use of chain extenders, much used in the field of polyester processing [28], can be fundamental. In some cases, the use of plasticizers with reactive groups having a low number of reactive groups per molecule (two or three) can allow better modulating PLA based compounds viscosity, thus also limiting the migration of the plasticizer from the final film thanks to its partial grafting to PLA [29]. The use of a compatibilizer bearing epoxide groups that decreases the melt flow rate of PLA based blends was also demonstrated to be effective for improving the processability by blown film extrusion [30]. On the other hand, the necessity of increasing the melt strength of pure PLA was yet investigated by several authors often using modifiers with epoxide moieties [31,32,33]. The crystallization of PLA is hindered by cross-linking [34,35] because of the decreased mobility of polymer chains. As the use of chain extenders having a low number of reactive groups per molecule do not result in extensive cross-linking, the capability of crystallizing of PLA is generally not widely affected [36] because a moderate branching also results in an increased crystallization velocity. In good agreement, Mallet et al. [37] combined an epoxydic chain extender with a nucleating agent consisting in talc and *N*,*N*′-ethylenbis (stearamide). They demonstrated that a higher kinetic of crystallization can also be achieved for chain extended and branched PLA formulated with adequate amounts of nucleating agents and plasticizers. Crystallization induced during process was also observed. Mechanical properties of the optimized PLA-based films obtained by blown extrusion revealed it to be a more flexible and ductile than pure PLA.

The blown film extrusion parameters were also demonstrated to influence the biaxially oriented PLA (BOPLA) film properties. In particular, Jariyasakoolroj et al. [38] evidenced that when the stretching rate and the draw ratio in biaxial-stretching technique, increased above a certain level, small δ-crystallites of about 10 nm size with isotropic orientation are mainly formed, thus improving the toughness of BOPLA film, which is about four times higher than the PLA film that is produced by conventional stretching. 

Although many papers evidence the high importance of producing PLA based films with improved resistance by blown extrusion, there is not a systematic work about the specific improvement of tear resistance as resulting from the modulation of composition in terms of reactive plasticizer and nucleating agents. In the present paper PLA based blends containing PBAT or PBS were employed for producing films by blown film extrusion. A reactive plasticizer was used in extrusion laboratory trials to both increasing flexibility and improve compatibility, then the process was scaled up in a semi-industrial extruder to prepare pellets adding also LAK 301 as nucleating agent. Films were then prepared by blown extrusion. The reactive plasticizer and the nucleating agent were used to achieve a good control of both processability and final mechanical properties of flexible films, with a focus on the tear resistance of films. The results are discussed by considering the morphological and mechanical properties of films that were prepared by blown extrusion.

## 2. Experimental Section

### 2.1. Materials

Poly(lactic acid) (PLA) containing 4,5% of D-lactic acid units, was PLA INGEO^TM^ 2003D from Natureworks LLC, (Minnetonka, MN, USA) having a nominal average molecular weight Mw = 199,590 and a density of 1.24 g/cm^3^. 

Polybutylene succinate (PBS) was PBS GS PLA^®^ FD92 WD from Mitsubishi Chemical Co., Tokyo, Japan. It consists of poly(butylenesuccinate-*co*-lactate) [39]. 

Poly(butylene adipate-*co*-terephthalate) (PBAT) was the product F Blend C1200 purchased from BASF, Ludwigshafen (FRG). Polypropyleneglycol diglygidyl ether (EJ) used as reactive plasticizer was EJ-400 Glyether^®^ Resin purchased from Jsi Co., Ltd., Pyeongtaek, Gyeonggi, Korea.

Dimethyl 5-sulfoisophthalate, potassium salt (LAK) used as nucleating agent was the product LAK 301, purchased from Takemoto Oil & Fat Co., Ltd., Gamagoori, Aichi, Japan.

### 2.2. Blends Preparation

Preliminary extrusion trials in a laboratory twin screw extruder were carried out to evaluate the capability of liquid plasticizer to improve PLA/PBAT or PLA/PBS flexibility and also compatibility (blends prepared: PLA/PBAT 67/33, PLA/EJ400/PBAT 67/10/23, PLA/PBS 67/33, PLA/EJ400/PBS 67/10/23). Then, a successive series of trials was carried out in semi-industrial extruder where the effect of nucleating agent onto mechanical and thermal properties was investigated (blends prepared: PLA/EJ400/PBAT/1%lak; PLA/EJ400/PBAT/2%lak; PLA EJ400/PBAT/3%lak; PLA/EJ400/PBAT/4%lak; PLA/EJ400/PBS/0%lak; PLA/PBS/EJ400/1%lak; PLA/PBS/EJ400/3%lak; PLA/PBS/EJ400/5%lak).

Regarding the blends prepared in miniextruder, all polymers (PLA, PBS, PBAT) in pellets were dried at 60 °C for 48 h, and they were mechanically mixed with a spatula in a glass Becker with liquid plasticizer at room temperature for 10 min. Then, the LAK was added and mixed again for 10 min using the same equipment. The mixtures were then processed with a MiniLab II HaakeTM Rheomex CTW 5 conical twin-screw extruder (Haake, Vreden, Germany). The extrusion was carried out at 190 °C with a screw speed of 100 rpm for a time of 1 min. The molten materials were transferred through a preheated cylinder to the Haake TM Minijet II mini injection moulder (Thermo Scientific, Waltham, MA, USA). The injection moulding of Haake type III specimens (Haake, Vreden, Germany).was carried out at 180 °C, with a holding time of 20 s and a pressure of 700 bar.

Before the extrusion in a semi-industrial twin screw extruder, after drying materials in pellets in an oven for 24 h, they were weighted and mixed mechanically at room temperature. Then, the extrusion was carried out in a Comac EBC 25 HT twin-screw extruder (Comac, Milan, Italy) to obtain granules. The temperatures in the zones 1 to 11 were: 190/175/180/170/185/185/185/185/180/160/165 °C, with the die zone at 140 °C. The screw rate was 200 rpm. The extruded strand is cooled in a water bath at room temperature and reduced in pellets by an automatic knife cutter. The granules, after drying, were filmed in an extrusion film blowing plant. The blown extrusion was carried out in a MAM40 equipment (MAM2, Varese, Italy) at 150 °C with a screw speed of 30 rpm, melt pressure 150 bar. The nip roll was running at the speed of 9 m/min.

The processing parameters investigated in the study are the blow-up ratio (BUR), the draw down ratio (DDR) and the forming ratio (FR). The BUR is the ratio of bubble diameter ($D_{b}$) to die diameter ($D_{d}$), defined by Equation (1).

(1) $$\mathrm{BUR}=\frac{D_{b}}{D_{d}}$$

where the bubble diameter can be evaluated from the lay flat width (LF), i.e., $D_{b}=2\cdot \frac{\mathrm{LF}}{\pi }$. The DDR and BUR are related as follows:

$$\mathrm{DDR}\cdot \mathrm{BUR}=\frac{\varepsilon_{0}}{\varepsilon_{f}}$$

where $\varepsilon_{0}$ is the die gap and $\varepsilon_{f}$ is the film thickness. The FR ratio provides an indication of the balance of the stretching between main direction, along the long axis of the bubble, (MD) and transverse (cross) direction, around the hoop direction of the bubble, (CD). FR is evaluated as follows

$$\mathrm{FR}=\frac{\mathrm{DDR}}{\mathrm{BUR}}$$

The blowing parameters are shown in Table 1, along with the processing parameters that are obtained from them.

### 2.3. Characterization

Differential scanning calorimetry (DSC) was performed by a Q200 differential scanning calorimeter from TA Instruments—Waters LLC, New Castle, DE (USA), equipped with a RSC cooling system and a nitrogen gas purge set at 50 mL/min flow rate was used for all of the calorimetric measurements. Aluminum pans with samples were sealed before measurement. The sample masses of the analyzed blown extruded films varied from 11 to 15 mg. Films were tested after 12 months from their production. They were heated from −50 to 250 °C at 10 °C/min. The specific melting enthalpy of the blown extruded films was calculated for PLA by summing the negative crystallization enthalpy ΔH_c_ and the positive melting enthalpy ΔH_m_ related to PLA transition. The ΔH values related to PLA or PBS were obtained by dividing per the weight fraction of PLA or PBS in the blend and multiplying per 100.The resulting value can be divided per the ΔH^0^_PLA_ of PLA with 100% crystallinity to obtain crystallinity value (111 J/g from the literature [40]). For PBS the crystallinity can be estimated by the specific melting enthalpy based on the melt blend baseline and dividing per 110.5 J/g as ΔH^0^_PBS_ [41]. 

Dynamical-mechanical analyses (DMA) were carried out using a Gabo Eplexor 100N (Netzsch Gabo Instruments GmbH, Ahleden, Germany) by heating at 2 °C/min from −100 °C to 200 °C. The tests were performed at a frequency of 1 Hz.

Tensile mechanical properties were measured using a 5500R tensometer (Instron, Norwood, MA, USA) with a load cell of 10 kN with software TestWorks 4.0 (MTS Systems Corporation, Eden Prairie, MN, USA) onto Haake type III specimens following the ASTM D638 indications. Samples were tested at two different temperatures. Dog bone samples were tested at room temperature at a crosshead speed of 10 mm/min. Samples in the form of thin strips with uniform width of 10 mm, total length of 100 mm and 0.05 mm thick, were tested at 25 °C, following the ASTM D882-12.

Trouser tear tests (ASTM D1938-02) were performed onto specimens of 2.5 cm width and 9 cm length with two legs having a width of 1.25 cm. The two legs were inserted in the grips at a distance of 5 cm. The test was performed at 250 mm/min.

The morphology of the blends was investigated by scanning electron microscopy (SEM) using a JEOL JSM-5600LV (Tokyo, Japan), by analysing cryofractured sample surfaces, previously sputtered with gold.

## 3. Results and Discussion

### 3.1. Reactive Plasticization of PLA/PBAT and PLA/PBS Blends by Using EJ400

**Figure 1:**
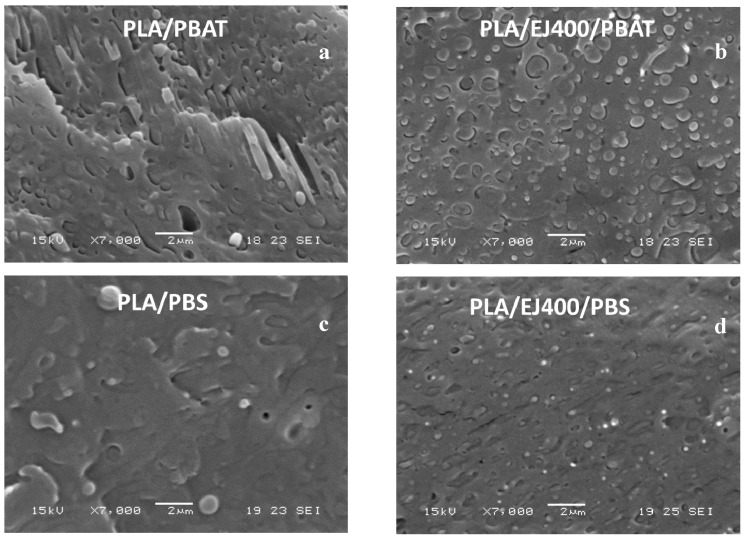
SEM micrographs onto cryogenic fractures samples of Poly(lactic acid) (PLA)/poly(butylene adipate-*co*-terephthalate) (PBAT) and PLA/poly(butylene succinate) (PBS) blends prepared in a laboratory extruder also in the presence of EJ400: (**a**) PLA/PBAT blend; (**b**) PLA/EJ400/PBAT blend; (**c**) PLA/PBS blend; (**d**) PLA/EJ400/PBS.

EJ400 is a reactive plasticizer, hence it modifies the mechanical properties of PLA/PBAT or PLA/PBS blends. Some preliminary extrusions carried out with the Haake miniextruder were then dedicated in understanding the effect of this additive. Hence tensile properties of injection moulded blends were preliminary determined to realize how the reactive plasticizer acts in the two systems. The shape of the stress-strain curves related to the different formulations is characterized by the presence of a maximum point (yield point) due to the necking of the specimen under tensile load. The yield stress thus corresponds to the maximum stress bearable by the material (tensile strength). The engineering value of the stress at break is lower than the yield stress. The results that are reported in Table 2 show that both the Young’s Modulus as well as the tensile stress at break are decreased by the addition of the plasticizer. The most evident result is in both systems the increase in elongation at break, due to the plasticization effect, but probably also to some improvement of compatibility between the two polymers. The morphology of the two blends (Figure 1) showed in fact relevant changes. 

In the case of PLA/PBAT blends extruded in the absence of EJ400, big elongated PBAT domains were observed, but the use of EJ400 allowed for the achievement of more homogeneous dispersed domains distribution inside the PLA matrix. In the case of PBS the use of EJ400 allowed for highly decreasing the domains dimensions in the sub-micrometric scale. The increase in interfacial area in both cases could be due to a reduction of interfacial tension, probably due to the formation of very low amount of PLA-PBAT and PLA-PBS copolymers thanks to EJ400 reactivity. In any case the change in viscosity of the two phases can also affect the morphology of the blends [42]. The change in viscosity in the melt of the two kinds of blends due to the addition of the EJ400 was almost negligible, as the torque values (Table 2) reveal. Usually, the addition of a liquid plasticizer results in a torque decrease [11]. For this reason, it can be tentatively hypothesized the occurrence of reaction between the epoxy group of EJ400 and the terminal groups of polyesters, both in the two separated phases and at the interface, resulting in general increase of molecular weight and also of viscosity.

### 3.2. Mechanical Properties of Plasticized and Nucleated Blown Extruded Films

The use of LAK was fundamental to obtain pellets from extrusion. In fact, the extrusion of PLA/PBAT blends in absence of LAK is difficult because the extruded strand after the cooling in water bath is still not rigid, hence the strand can not be reduced in pellets by the cutter. Inside the strand, having a thickness of about 3–5 mm, the material cools slowly and is still amorphous above its glass transition, hence in a rubbery state in proximity of the cutter. On the other hand, when LAK is added in these blends, the preparation of pellets is possible and reliable. This behaviour can be explained by considering the nucleating effect of LAK. In plasticized PLA, the crystallinity developed during rapid cooling in injection moulding, in the range 0.3–7.3%, could be increased by adding LAK and showed a maximum for a LAK content of 3% [27]. The presence of LAK was demonstrated to be effective for increasing the crystallinity in the early cooling stage, thus rapidly increasing the material rigidity making it easy to cut. The prepared formulations with a different LAK content were prepared to verify if the content of LAK also has an effect on the blowing extrusion of films and on their properties. Regarding the processing in blowing extrusion, all the formulations resulted suitable to be processed in the same conditions (Table 1) and the process resulted reliable. The stress-strain curves related to films do not show any points of maximum. This agrees with the observed behaviour during the tensile tests, not showing the occurring of necking. For this reason, the maximum value of stress bearable by the material corresponds to the stress at break.

**Figure 2:**
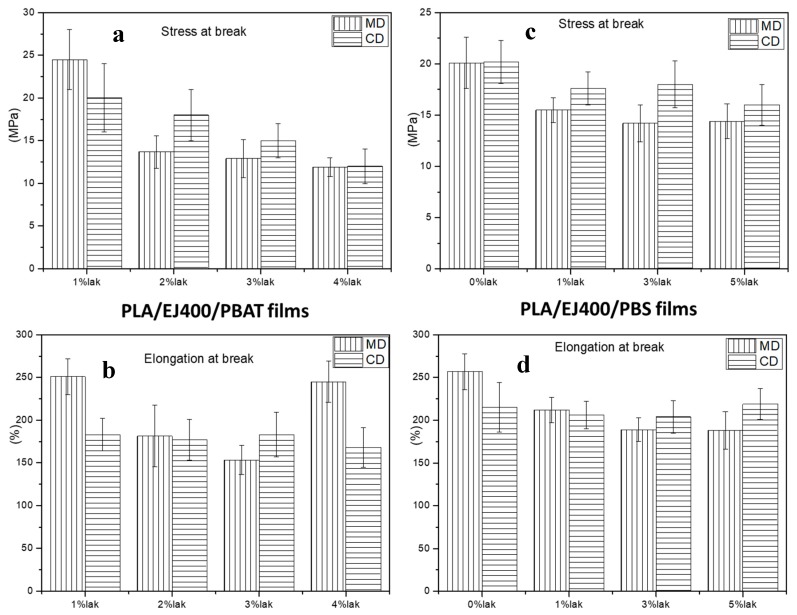
Tensile tests related to blown extruded films: (**a**) Stress at break of PLA/EJ400/PBAT films; (**b**) Elongation at break of PLA/EJ400/PBAT films; (**c**) stress at break of PLA/EJ400/PBS films; (**d**) Elongation at break of PLA/EJ400/PBS films

The results of the tensile tests are reported in Figure 2.

By examining the results, it is possible to observe that by increasing the percentage of LAK both in the case of mixtures based on PLA/PBAT and PLA/PBS, there is a reduction of the tensile stress at break. This can be attributed to the tendency of LAK to form agglomerates (as it will be shown in morphologic characterization), resulting as stress concentrator points and favouring the failure. In any case, it can be useful to investigate, by considering both tensile (Figure 2) and DMTA results (Table 3 and Table 4).

Regarding the PLA/PBS films, it is evident that at low deformation (DMTA data), the elastic modulus in CD direction is higher than in the MD direction. Accordingly, stress at break is higher in CD than in MD direction. The results show that the macromolecules are preferentially oriented in the CD direction. The differences in the two directions are less evident by increasing the content of LAK. Hence, in this case, the film is more isotropic in orientation and this could be ascribed to the presence of LAK influencing the local morphology, both by its presence in micro-particles and because of its influence on crystallinity. 

In the case of PLA/PBAT film, the behavior at low deformation is similar to PLA/PBS films: an orientation in CD direction can be reasonably hypothesized, but it depends on the LAK content, being absent in the case of high content (3 and 4 wt. %).

In the case of behaviour at break, the values are higher in the MD direction at 1% and 4% of LAK, whereas they are higher in the CD direction in the other compositions. This result can be due to the specific tensile behavior of these films, allowing, when a deformation is applied during the tensile test, a better deformation. The low and high content of LAK is correlated to a slighly higher cristallinity content than the other two films (Table 5). These crystals are probably responsible of increasing the resistance of the film more in the MD direction.

In general, in both PLA/PBS and PLA/PBAT films, the increase of LAK content leads to a decrease in anisotropy of films. 

**Figure 3:**
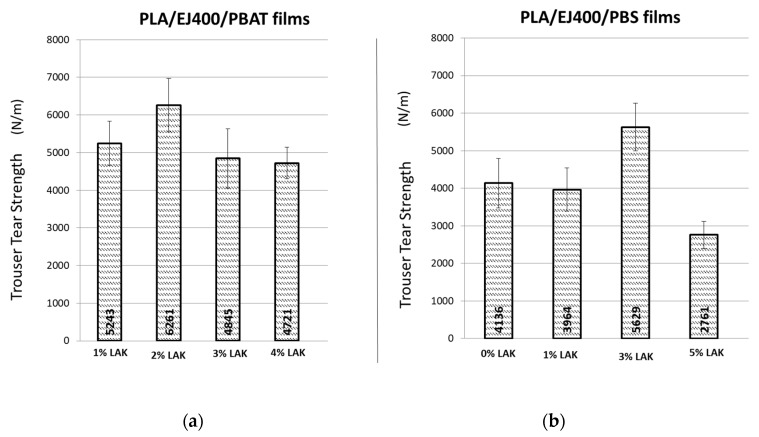
Trouser tear tests related to blown extruded film: (**a**) PLA/EJ400/PBAT films; (**b**) PLA/Ej400/PBS films.

Trouser tear tests were also performed onto the blown extruded films. The tests performed in the MD direction showed no deformation of trouser specimens legs. The tests performed on CD direction showed the rapid rupture following the MD direction. This behaviour is in agreement with a reduced resistance in this direction and suggested that the tearing properties should be controlled uniquely in the direction where less orientation is obtained. The results (Figure 3) showed that the tear resistance depends on LAK content, and that, in both kinds of blends, an optimal content of LAK can be found. In PLA/PBAT blends, it corresponds to 2% of LAK, whereas in PLA/PBS blends, it corresponds to 3% by weight of LAK. Interestingly, it can be found that the tear resistance of polypropylene films, determined by tests made in the same conditions, resulted in a value of 5560 N/m. Thus, some of the PLA based films showed a tear resistance higher than polypropylene films. As the tear resistance is considered the main drawback of PLA based films, these results seem quite useful for overcoming this issue. Anyway, it is important to notice that polypropylene is not considered a material with a high tear resistance in the production of commercial blown films, whereas low density polyethylene (55,560 N/m) [43] shows better properties and should be considered a reference in view of developing an effective biobased tear resistant film. 

As yet evidenced, observing the data in Table 3 and Table 4 related to both PLA/PBAT and PLA/PBS based blends, it can be noticed that the storage modulus value determined on specimen on CD direction is considerably higher than the one in MD. Interestingly, the Tg values for both polymers resulted slightly lower in MD than in CD direction, in according with a lower mobility in the preferential orientation direction of polymer chains. The glass transition temperatures (Tgs) of both PLA and PBAT or PBS decrease in both blends with respect to the values that are reported for pure polymers. In fact, for PLA, PBAT, and PBS glass transition temperature of 60 °C, −27 °C, and −33 °C were reported [44,45]. This agrees with the plasticizing effect of EJ400. The presence of LAK does not significantly affect the glass transition values.

### 3.3. Thermal and Morphological Features of Plasticized and Nucleated Blown Extruded Films

**Figure 4:**
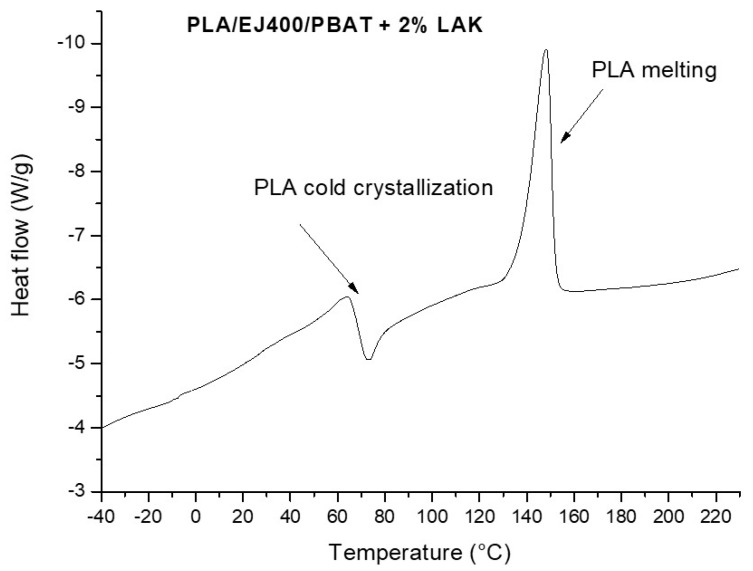
Heating thermogram of a PLA/EJ400/PBAT blown extruded film containing dimethyl 5-sulfoisophthalate, potassium salt (LAK).

The heating thermogram of a PLA/EJ400/PBAT (Figure 4) shows the crystallization of PLA occurring above the glass transition and the successive melting peak. The specific melting temperature values show that the total crystallinity of these films is quite similar in all the formulations. Calculations allowed to estimate that it ranges between 18% and 20%. It means that during the processing a significant amount of crystals was formed in the presence of LAK, but this amount seems only slightly dependent on LAK content. The other thermal characteristics, such as *T_c_* and *T_m_* did not significantly change as a function of LAK content (Table 5).

**Figure 5:**
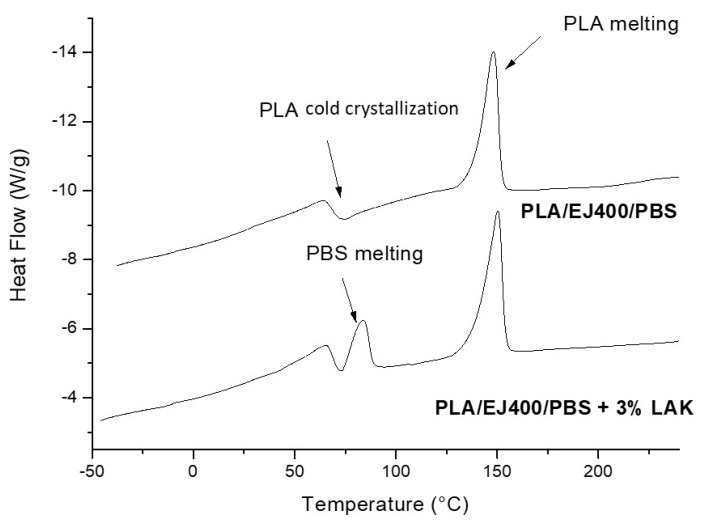
Heating thermogram of a PLA/EJ400/PBS blend and of a PLA/EJ400/PBS blend containing LAK.

The heating thermogram of PLA/EJ400/PBS blend without LAK showed the transitions that are typical of PLA: the cold crystallization and the melting (Figure 5). The crystallinity of PLA was 21.4% but PBS resulted amorphous, as the melting peak could not be seen in the thermogram. The situation completely changed in the presence of LAK. In fact, in this case, also the peak of PBS can be observed in the thermogram. Hence, in the presence of LAK, we obtained the nucleation of PBS. On the other hand, the values of crystallinity also increased for PLA. The LAK thus behaved as a nucleating agent for both PLA and PBS. No significant changes in melting temperatures can be observed as a function of LAK content (Table 6).

The DSC characterization showed the presence of significant amount of crystals in the blend films, but it could not allow for understanding the maximum like trend observed for tearing strength. 

**Figure 6:**
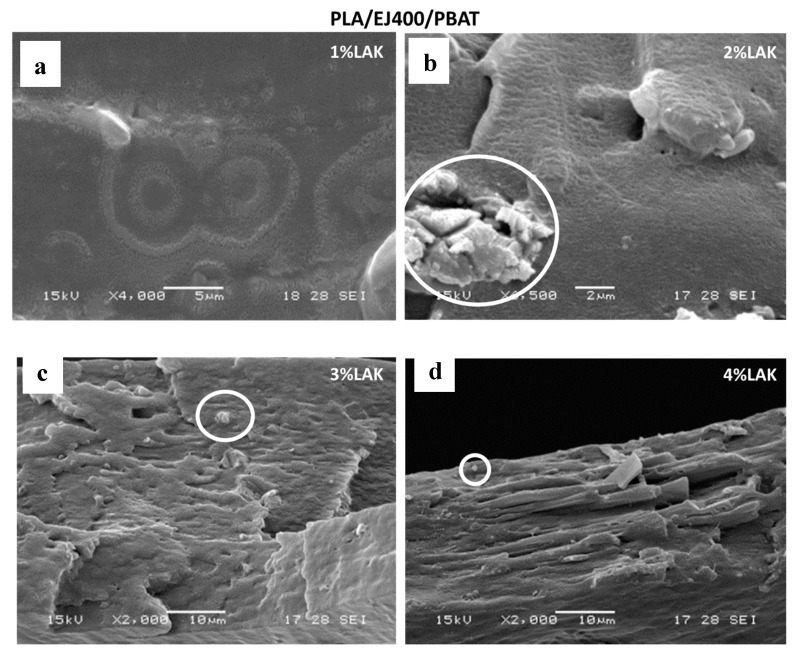
SEM micrographs of cryogenic fractures films based on PLA/EJ400/PBAT containing different amount of LAK: (**a**) 1% LAK; (**b**) 2% LAK; (**c**) 3% LAK; (**d**) 4% LAK.

**Figure 7:**
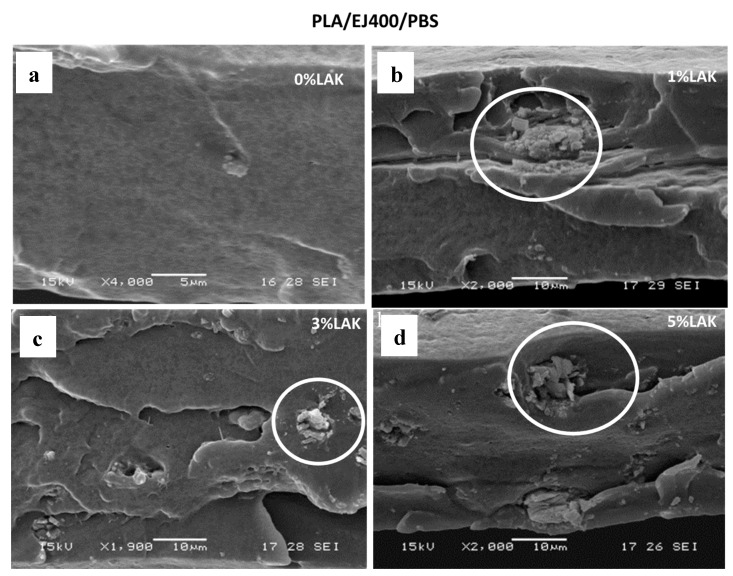
SEM micrographs of cryogenic-fracture films based on PLA/EJ400/PBS containing different amount of LAK: (**a**) 0% LAK; (**b**) 1% LAK; (**c**) 3% LAK; (**d**) 5% LAK.

The morphology of blown extruded films was investigated by SEM. In general, the films consisted of a biphasic system with good adhesion between the phases in good agreement with morphology observation related to blends prepared in miniextruder (Figure 1). PBAT domains elongated in the preferential direction (DC) can be noticed in the films (Figure 6). On the contrary, PBS domains (Figure 7) cannot be distinguished easily, probably because of the lower dimension, sub-micrometric, and the very good adhesion to the PLA matrix.

In both blends, some aggregates with sharp edges attributable to the presence of the nucleating agent, can be occasionally observed during the analysis, and it is more frequent in blends containing higher LAK amount. The presence of these agglomerates can explain the slightly lower value of elongation at break and tensile strength in films obtained with a higher content of LAK. The maximum like trend of tearing strength can be also explained by considering that the presence of aggregates can reduce the capacity of the material to dissipate energy during tearing tests because of premature breaking.

## 4. Conclusions

After a preliminary study showing the effective plasticizing and compatibilizing effect of EJ400 in PLA/PBAT and PLA/PBS blends, granules based on PLA/EJ400/PBAT and PLA/EJ400/PBS were prepared in a semi-industrial extruder. The addition of a nucleating agent (LAK) allowed for a good control of the extrusion process.

Flexible films were then prepared by blown film extrusion by using the prepared granules.

The tensile and dynamical-mechanical properties as well as tearing resistance were studied as a function of content of LAK. The films prepared with both kinds of blends showed tensile strength in the range 12–24 MPa and elongation at break in the range 150–260%. The preferential orientation of macromolecules in the CD direction resulted in a higher Elastic Modulus, determined by DMTA, in this direction, but in both PLA/PBS and PLA/PBAT films was less evident for high content of LAK. The best tearing performances were obtained in the blend PLA/PBAT with 2% of LAK and blend PLA/PBS with 3% of LAK. Hence, the use of LAK in PLA based blends in blown extrusion resulted important for a thorough modulation of mechanical properties. In fact, a significant amount of crystalline fraction was generated in the films.

The tearing resistance is higher than the one of PP but still much lower than the one of LDPE [43].

## Figures and Tables

**Table 1:** Blown extrusion parameters.

Blown Extrusion Parameters	Values
Layflat width	410 mm
Bubble diameter	261 mm
Die diameter	55 mm
Die gap	0.8 mm
Film thickness	0.05 mm
Blow-up ratio BUR	4.75
Draw down ratio DR	3.37
Forming ratio FR	0.71

**Table 2:** Tensile tests performed on laboratory extruded PLA/PBAT and PLA/PBS blends by using EJ400 as reactive plasticizer.

Composition	Final Torque (N·cm)	Young Modulus (GPa)	Tensile Strength (MPa)	Elongation at Yield (%)	Stress at Break (MPa)	Elongation at Break (%)
**PLA/PBAT** **67/33**	73	2.6 ± 0.2	38.8 ± 3.1	1.4 ± 0.5	31 ± 3	110 ± 20
**PLA/EJ400/PBAT** **67/10/23**	69	1.4 ± 0.1	28.8 ± 0.9	1.2 ± 0.2	29 ± 2	140 ± 10
**PLA/PBS** **67/33**	58	1.8 ± 0.2	45 ± 1	1.3 ± 0.5	32 ± 5	100 ± 20
**PLA/EJ400/PBS** **67/10/23**	55	2.7 ± 0.2	37 ± 2	0.9 ± 0.1	30 ± 4	150 ± 40

**Table 3:** DMTA results about blends based on PLA/EJ400/PBS.

LAK (wt. %)	MD			CD		
	E’ (GPa)	Tg_PLA_ (°C)	Tg_PBS_ (°C)	E’ (GPa)	Tg_PLA_ (°C)	Tg_PBS_ (°C)
0	2.9 ± 0.1	35.1 ± 0.9	−30 ± 2	4.2 ± 0.2	37 ± 2	−31 ± 3
1	3.1 ± 0.3	32 ± 2	−38.1 ± 0.9	3.4 ± 0.1	37.2 ± 0.7	−33 ± 2
3	2.2 ± 0.1	33 ± 2	−38 ± 2	2.2 ± 0.3	39 ± 1	−31 ± 3
5	1.8 ± 0.2	31 ± 1	−36 ± 3	2.6 ± 0.2	33.2 ± 0.9	−33 ± 1

**Table 4:** DMTA results about blends based on PLA/EJ400/PBAT.

LAK (wt. %)	MD			CD		
	E’ (GPa)	Tg_PLA_ (°C)	Tg_PBAT_ (°C)	E’ (GPa)	Tg_PLA_ (°C)	Tg_PBAT_ (°C)
1	2.4 ± 0.2	35 ± 2	−37 ± 1	4.1 ± 0.3	36.9 ± 0.7	−36 ± 2
2	2.1 ± 0.1	35 ± 1	−38 ± 2	5.3 ± 0.4	37 ± 2	−33 ± 1
3	3.2 ± 0.3	35.1 ± 0.8	−33 ± 2	3.4 ± 0.2	35 ± 1	−33 ± 3
4	2.6 ± 0.1	35 ± 2	−33.8 ± 0.7	2.2 ± 0.2	35.1 ± 0.9	−33 ± 1

**Table 5:** DSC data related to PLA/EJ400/PBAT films.

% of LAK	ΔH_c_ (J/g)	ΔH_m_ (J/g)	Total ΔH_PLA_^a^ (J/g)	T_c_ (°C)	T_m_ (°C)
1	7.681	22.48	22.09	73.67	147.38
2	9.482	22.60	19.53	73.46	148.28
3	8.557	22.38	20.65	70.78	147.62
4	8.335	23.42	22.53	72.95	149.07

^a^ specific melting enthalpy = ΔH_m_ − ΔH_c,_ with values corrected keeping into account the content of polymers in the blends. It is a relative indicator of crystallization degree.

**Table 6:** DSC data related to PLA/EJ400/PBS films.

% of LAK	ΔH_cPLA_^b^ (J/g)	ΔH_mPBS_ (J/g)	ΔH_mPLA_^b^ (J/g)	Total ΔH_PBS_^a^ (J/g)	Total ΔH_PLA_^a^ (J/g)	T_cPLA_ (°C)	T_mPBS_ (°C)	T_mPLA_ (°C)
0	7.198	-	23.27	-	28.42	74.75	-	148.22
1	0.6645	7.626	24.96	37.24	34.52	73.70	84.29	148.93
3	0.9190	7.067	23.53	34.48	32.08	74.87	84.18	150.29
5	0	5.968	25.73	29.06 ^c^	35.63	-	83.90	148.92

^a^ specific melting enthalpy = ΔH_m_ − ΔH_c,_ a relative indicator of crystallization degree; ^b^ The value was calculated by integration but is slightly underestimated because of the superposition of the melting peak of PBS (Figure 5); ^c^ The value was calculated by integration but is underestimated, due to the superposition of signals.
